# Adsorption and anion exchange insight of indigo carmine onto CuAl-LDH/SWCNTs nanocomposite: kinetic, thermodynamic and isotherm analysis†

**DOI:** 10.1039/c8ra09562k

**Published:** 2019-01-02

**Authors:** Noha Almoisheer, F. A. Alseroury, Rajeev Kumar, M. Aslam, M. A. Barakat

**Affiliations:** Department of Physics, Faculty of Science, King Abdulaziz University Kingdom of Saudi Arabia; Department of Environmental Sciences, Faculty of Meteorology, Environment and Arid Land Agriculture, King Abdulaziz University Jeddah 21589 Kingdom of Saudi Arabia mabarakat@gmail.com +96626952364 +96626400000 ext. 64821; Center of Excellence in Environmental Studies, King Abdulaziz University Kingdom of Saudi Arabia; Central Metallurgical R & D Institute Helwan 11421 Cairo Egypt

## Abstract

Two-dimensional layered materials are gaining much attention in the field of wastewater purification. Herein, we report the synthesis and characterization of an anion selective copper–aluminum-layered double hydroxide/single-walled carbon nanotubes (CuAl-LDH/SWCNTs) composite for the scavenging of organic dye indigo carmine (IC) from aqueous solution. A facile urea hydrolysis method was used for the controlled growth of the metal hydroxides over the SWCNTs. Structural characterization of the prepared materials was investigated using X-ray photoelectron spectroscopy (XPS), scanning electron microscopy (SEM), and X-ray diffraction (XRD) techniques. The obtained results revealed that the CuAl-LDH/SWCNTs composite has a higher potential for the removal of IC in comparison to CuAl-LDH and SWCNTs. The enhanced adsorption capacity of the composite revealed that deposition of CuAl-LDH over SWCNTs increases the active adsorption sites and promotes the interactions between the composite and IC dye *via* anion exchange, electrostatic, π–π, hydrogen bonding *etc.* Moreover, adsorption kinetics, isotherms, and thermodynamic studies have been also proposed to illustrate the mechanism of the IC adsorption onto the CuAl-LDH/SWCNTs composite. Thermodynamic parameters showed that the adsorption of IC dye onto the CuAl-LDH/SWCNTs composite was exothermic and spontaneous in nature. Intra-particle diffusion was determined to be the rate-limiting step and adsorption of IC followed the Langmuir isotherm model with the maximum monolayer adsorption capacity 294.117 mg g^−1^ at 20 °C. The results suggest that the CuAl-LDH/SWCNTs composite is a potential material for IC adsorption in aqueous solution.

## Abstract 2

Two-dimensional layered materials are gaining much attention in the field of wastewater purification.

## Graphical abstract

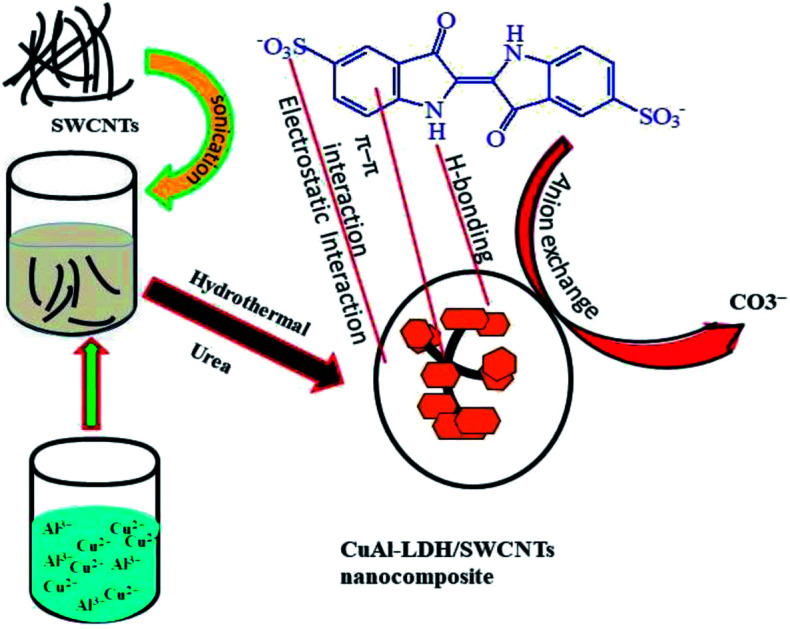

## 1. Introduction

The application of various types of dyes and pigments in a variety of industries such as the textile, cosmetics, plastics, paper, printing *etc.* industries, releases a large amount of colored substances into wastewater streams.^1–4^ Wastewater effluents containing dyes cause major problems in the environment due to their color and their breakdown products.^5^ The toxic nature of dyes may cause allergies, skin irritation and even are carcinogenic.^6,7^ Indigo carmine (IC) is an anionic dye and used in various industries such as the textile, food, and cosmetics industries. IC is a highly toxic dye which causes irritation to the eye, respiratory tract and skin.^7,8^ Due to their toxicity, it is necessary to remove dyes from contaminated water before discharging to the environment. There are different techniques used to treat wastewater, such as coagulation/flocculation, ozone treatment, chemical oxidation, membrane filtration, ion exchange, photocatalytic degradation, and adsorption.^3,4,9^ The adsorption processes have proven to be one of the best treatment methods because of their high efficiency, low price, easy operation and handling.^1,4,6,9,10^ Different types of natural and synthetic adsorbents have been used to treat wastewaters containing dyes including CNTs,^1,5^ agricultural waste,^2,4^ graphene oxide,^6^ activated carbons,^11^ zeolite,^12^ clay,^13^ polymers^7,14^ and their composites. However, disadvantages of these adsorbents are low adsorption capacities and separation inconvenience. Hence, there is a need to investigate new promising adsorbents having high adsorption capacity in a short time.^14^ Recently, the growth of nanotechnology has improved water treatment technology by representing the suggestion that many water quality issues could be resolved or greatly ameliorated using nanotechnology products.^1,6,15^

Since the discovery, carbon nanotubes (CNTs) attracts the attention of researchers around the world due to their excellent properties and applications in a variety of fields.^1,5,16,17^ The application of the CNTs in the field of wastewater purification as an adsorbent has been explored and a large number of the articles have been published which are revealing its potential as an excellent adsorbent due to a large specific surface area and tubular structure.^1,16,17^ However, expensive cost, separation from the aqueous solution and their hydrophobic surface may limit the CNTs application as an adsorbent.^1,5,16,17^ In recent year, the disadvantages related to CNTs can be conquering through making composites with polymers, chitosan, activated carbon fabric, graphene, metal oxides *etc.*^1,18–22^

In last decade, two-dimensional nanomaterials such as graphene oxide, graphitic carbon nitride, boron nitride, metal-layered double hydroxide *etc.* are gaining the much attention due to higher stability, large surface area, high adsorption capacity and reusability.^22^ Metal-layered double hydroxide has attracted huge attention due to its applications such as anion exchanger, catalysis, photochemistry, electrochemistry, environmental applications and so forth.^23,25^ Metal-layered double hydroxide including hydrotalcite and hydrotalcite-like are a versatile class of ionic clay.^24–26^ Metal-layered double hydroxide has the general formula M_*a*_^2+^M_*b*_^3+^(OH)_+2*a*+2*b*_^−^(X^−^)·*x*H_2_O, where two kinds of metal ion, M^2+^ is a divalent metal cation and M^3+^ as trivalent metal cation, forming the layered structure together with hydroxyl groups, an interlayer anion, and water molecules. The interlayer anions can be exchanged between layers where the H_2_O molecules are placed.^25–28^ Recently, the use of the metal-layered double hydroxide as an adsorbent has been investigated by several researchers. Ahmad *et al.*,^8^ synthesized the Mg/Fe-LDH for the removal of IC dye. The adsorption of phosphate by biochar composite with Mg–Al and Mg–Fe-LDH was investigated by Wan *et al.*^29^ The authors reported that LDH based biochar composite showed the fast and efficient removal of the phosphate ions. Sepehr *et al.*,^30^ used the Mg/Al-LDH for the removal of metronidazole antibiotic form the aqueous solution and the maximum adsorption capacity was 62.804 mg g^−1^. Previous studies showed that LDH are the good adsorbents for the removal of wastewater contaminants.^8,29–31^ Due to the presence of exchangeable anions and 2D layered structure, metal-layered double hydroxide based materials are the good adsorbent for the removal of anionic pollutants.^8,29,30^ Given the various attractive and anion selective features of the metal-layered double hydroxide, we hypothesized that if we decorate them onto the SWCNTs, then we can combine the feature of both materials into the single phase to enhance the IC scavenging properties.

Herein, synthesis of CuAl-LDH/SWCNTs nanocomposite for the scavenging of anionic IC molecules has been investigated. The prepared CuAl-LDH/SWCNTs nanocomposite showed highly selective behaviors with high adsorption capacity for IC molecules. Moreover, different kinetics and isotherm models are also fitted to the experimental data to investigate the adsorption mechanism.

## 2. Materials and method

### 2.1. Materials

Indigo carmine dye, molecular formula C_16_H_8_N_2_Na_2_O_8_S_2_, color index = 73 015, molecular mass as 466.36 g mol^−1^ and maximum light adsorption *λ*_max_ = 610 nm was purchased from Merck. Single-walled carbon nanotubes (SWCNTs) synthesized using CVD method were purchased from Beijing Deke Daojin Science and Technology Co., Ltd, China, with an average 1.1 nm diameter, surface area 450 m^2^ g^−1^ and the purity were above 95%. The length of these tubes was in the range of 5–30 μm.

### 2.2. Synthesis of CuAl-LDH/SWCNTs composite

For the synthesis of CuAl-LDH/SWCNTs nanocomposite, a hydrothermal method was used. Initially, 1.23 g SWCNT was suspended in 50 ml of deionized water and then solubilized by sonication for 1 h. The used salts for copper and aluminum were Cu(ii) or Al(iii) salts used were CuSO_4_ and Al(NO_3_)_3_. Thereafter, 25 ml of each, 0.05 M Cu(ii) and 0.0166 M Al(iii) were added into the SWCNTs solution. After vigorous stirring for 30 min, 25 ml of 0.25 M urea was added and continuously stirred for 1 h. The resulting mixture was transferred to a Teflon lined hydrothermal autoclave reactor. The reactor was heated at 140 °C for 18 h and left the reactor until cool down to room temperature. The obtained product was filtered, thoroughly washed with de-ionized water, acetone and then dried at 105 °C for 24 h. The pure CuAl-LDH material was also synthesized using the same method without adding SWCNTs.

### 2.3. Characterizations

The surface morphology of pure SWCNTs and CuAl-LDH/SWCNTs nanocomposite was studied by (FE-SEM) (JSM – 7600F; JEOL – Japan). XRD patterns of pure SWCNTs and CuAl-LDH/SWCNTs nanocomposite were analyzed by X-ray diffractometer (Ultima-IV; Rigaku, Japan) operating at 40 kV and 40 mA over the range (2*θ*) of 10–80. The Cu Kα X-ray source with a wavelength of 0.154056 nm was used. The X-ray photoelectron spectroscopy (XPS) spectra of CuAl-LDH/SWCNTs was recorded with a (SPECS GmbH, Germany) spectrometer using a non-monochromatic Mg-Kα (1253.6 eV) X-ray source (100 W) with 13.5 kV at 10^−10^ mbar base pressure.

### 2.4. Adsorption analysis

The adsorption properties of the synthesized materials were investigated in a batch process using 20 ml IC solution of fix concentrations and at constant temperatures. A fixed amount of adsorbent (10 mg) was used and the pH of the IC solutions was varied from 2 to 10. The desired pH of the solution was adjusted using 0.1 M HCl and 0.1 M NaOH solutions. The adsorption equilibrium time studies were performed at the reaction time ranges from 0 to 360 min at fix pH and concentration. The isotherm and thermodynamics studies were performed at the temperature varied between 20 to 40 °C and IC solution concentrations range from 50 to 500 mg L^−1^. UV-visible spectrophotometer (UV-1800, Shimadzu, Japan) was used for determination of initial and residual IC concentration in the solutions. The adsorption capacity of the prepared adsorbents was determined by the following equation1
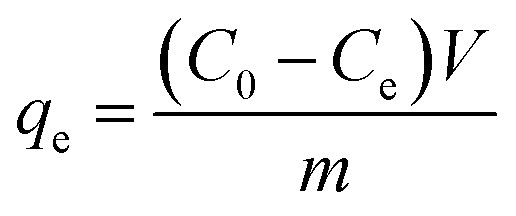
where *C*_0_ and *C*_e_ are the initial and remaining IC dye concentration in solution (mg L^−1^), respectively in time *t*. *q*_e_ represent adsorption capacity (mg g^−1^). *V* and *m* are the sample volume in (L) and adsorbent mass in (g).

## 3. Results and discussion

### 3.1. Synthesis and characterization of CuAl-LDH/SWCNTs nanocomposite

**Fig. 1:**
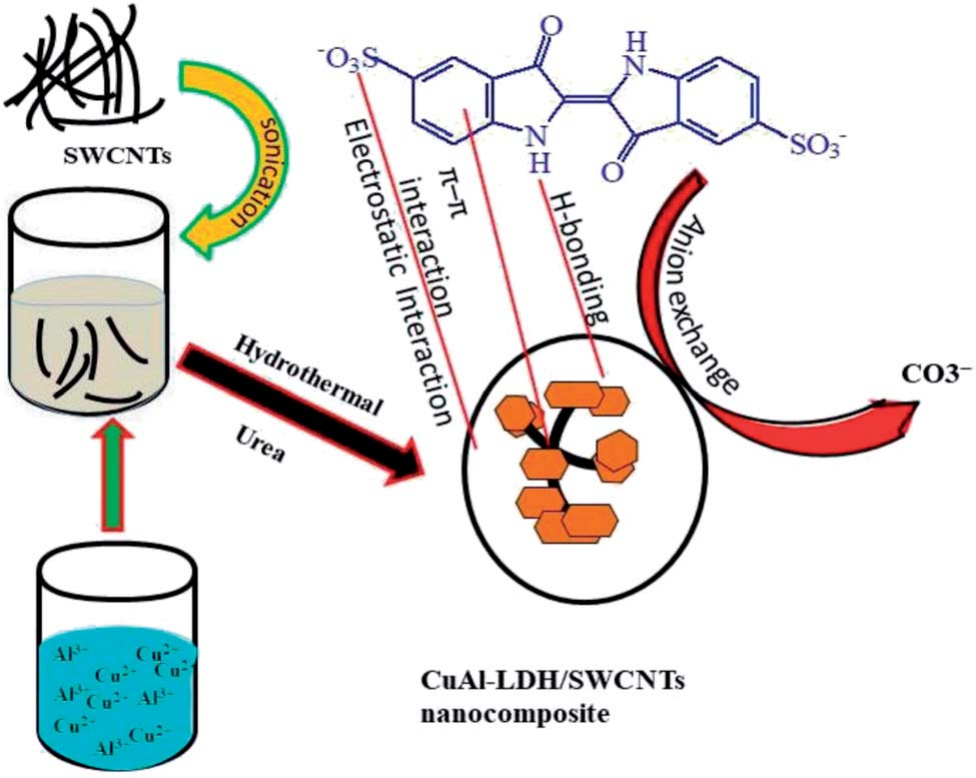
A schematic diagram for the synthesis of CuAl-LDH/SWCNTs nanocomposite.

The synthesis of the CuAl-LDH/SWCNTs nanocomposite is performed by the urea hydrolysis method under the hydrothermal conditions as shown in Fig. 1. The urea releases the carbonate and hydroxides upon heating which form the monodisperse hydrotalcite particles in the presence of Cu^2+^ and Al^3+^ with a good crystallinity degree and form the metal hydroxides.^32^

**Fig. 2:**
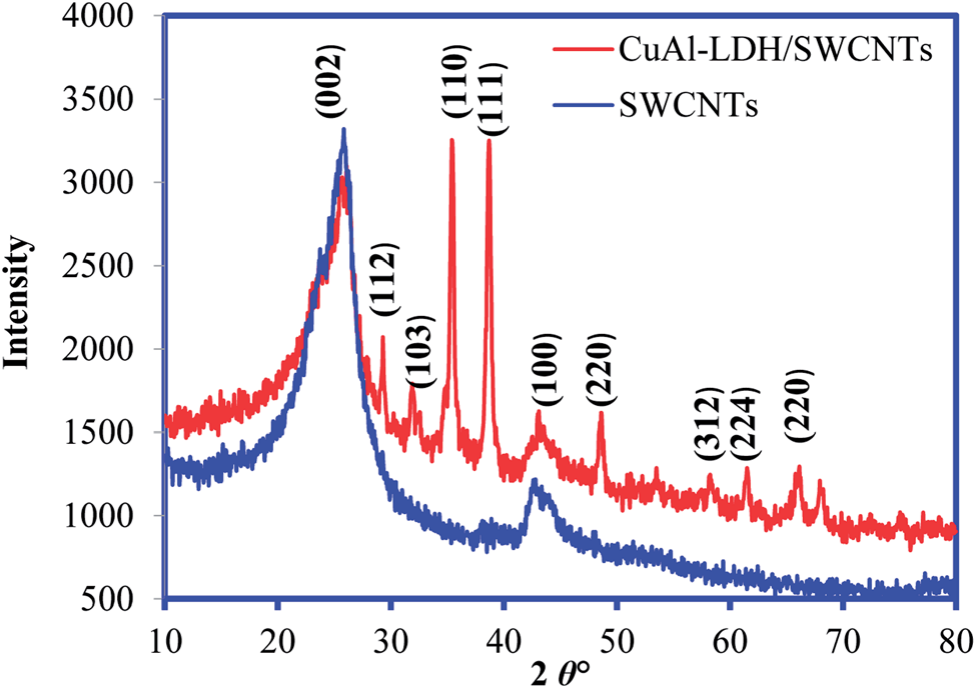
XRD pattern of SWCNTs and CuAl-LDH/SWCNTs nanocomposite.

The crystallinity and structure of both SWCNTs and CuAl-LDH/SWCNTs composite were recorded by XRD as depicted in Fig. 2. The diffraction pattern of pure SWCNTs showing the peaks at 2*θ*° – 25.8 and 42.55 which correspond to diffraction of the (002) and (100) of graphitic planes of the SWCNTs. The XRD pattern of CuAl-LDH/SWCNTs showed the characteristic peaks for the graphitic carbon at the almost same position and also showed the sharp peaks revealing the crystallinity and reflections for the deposited CuAl-LDH structure. These reflections are the characteristics of LDH in like hydrotalcite phase.^27^

The SEM images of SWCNTs and CuAl-LDH/SWCNTs composite are shown in Fig. S1.† A significant change in the morphology of SWCNTs (Fig. S1a†) can be seen in the SEM image after the decoration of CuAl-LDH (Fig. S1b†). The pure SWCNTs are showing the bundle like structure (Fig. S1a†) while SEM image (Fig. S1b†) of CuAl-LDH/SWCNTs composite showing the successful deposition of the CuAl-LDH onto SWCNTs. Moreover, to confirm the presence of the CuAl-LDH in the composite, XPS analysis is performed.

**Fig. 3:**
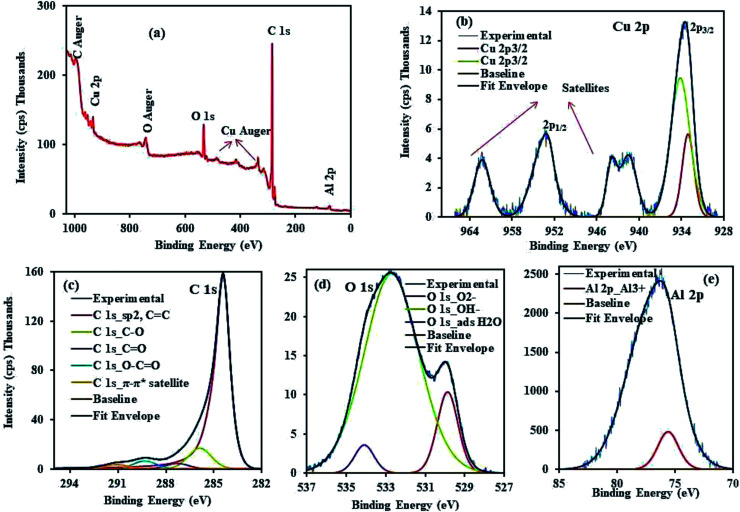
XPS analysis of CuAl-LDH/SWCNTs nanocomposite (a) survey, and high resolution spectra of (b) Cu 2p (c) C 1s (d) O 1s and (e) Al 2p.

The XPS analysis of CuAl-LDH/SWCNTs composite was performed to determine the elemental composition and oxidation state of the individual elements in the composite. Fig. 3a showing the XPS survey spectrum demonstrates the elemental composition of CuAl-LDH/SWCNTs which revealing the % atomic concentration of C 1s (86.168%), O 1s (12.172%), Cu 2p (1.112%) and Al 2p (0.548%) at the binding energy of 284.45, 532.75, 933.15 and 76.15 eV, respectively. The deconvoluted high resolution spectra (Fig. 3b) of Cu 2p shows the peaks at 933.05 eV (individual Cu^2+^ centers), 934.15 eV (clustered Cu^2+^ species) corresponding to Cu 2p_3/2_ and while the peak at 952.75 eV belongs to Cu 2p_1/2_.^27^ The main C 1s peak in Fig. 3c located at 284.45 eV is assigned to sp^2^ carbon from SWCNTs. Also, there were relatively weak peaks located at 285.9 eV, 287.3 eV and 289.35 eV corresponding to the C–O bond, C=O bond, and O–C=O bond, respectively.^33^ The XPS spectrum of O 1s (Fig. 3d) shows a strong peak corresponding to the O–H bond of the carboxyl group on the SWCNT surface.^34^Fig. 3e shows a high-resolution spectrum of Al 2p appears at the binding energy 75.6 eV which indicate the presence of Al^3+^ in Al–OH.^35^

### 3.2. Indigo carmine adsorption studies

#### 3.2.1. Effect of solution pH and adsorption mechanism

**Fig. 4:**
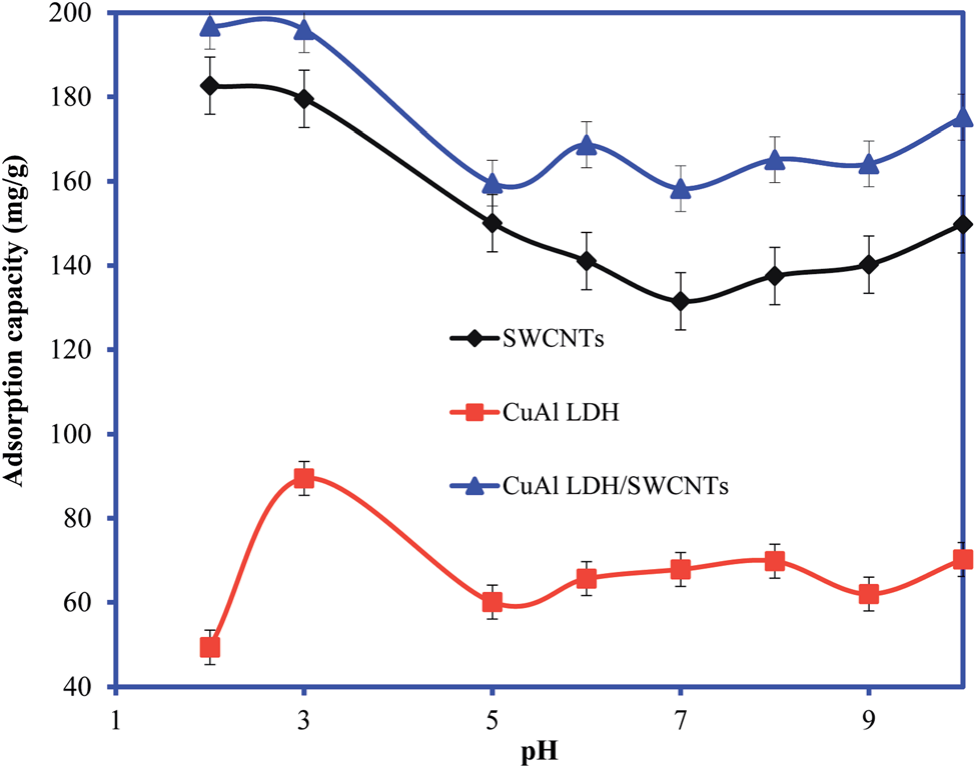
Plot for the adsorption of IC molecules onto CuAl-LDH, SWCNTs and CuAl-LDH/SWCNTs nanocomposite at varying solution pH. (Conc. 100 mg L^−1^, *V* 20 ml, temp. 20 °C, time 6 h, *m* 0.01).

The solution pH is considered one of the main factors which control the interaction between the adsorbent and adsorbate. The uptake of the IC molecules by the CuAl-LDH, SWCNTs and CuAl-LDH/SWCNTs nanocomposite at the different solution pH is shown in Fig. 4. The results show that adsorption capacity order: CuAl-LDH < SWCNTs < CuAl-LDH/SWCNTs for IC at all the studied pH. The results revealed that the acidic solution conditions are favorable for the removal of IC molecules and optimum adsorption is observed at pH 2.^36^ The adsorption capacity of CuAl-LDH/SWCNTs nanocomposite for IC is 196.78 mg g^−1^ which is evidently greater than that of both SWCNTs (179.5 mg g^−1^) and CuAl-LDH (89.46 mg g^−1^). The higher adsorption of the IC onto the CuAl-LDH/SWCNTs nanocomposite can be explained on the basis of the hybrid nature of the composite which has the properties of the CuAl-LDH and SWCNTs and having a large number of the functional groups compared to the CuAl-LDH and SWCNTs. The adsorption of IC onto all studies adsorbents, CuAl-LDH, SWCNTs and CuAl-LDH/SWCNTs decreases with the increase in the solution pH. The changes in the IC uptake behavior at varying solution pH can be explained on the basis of ion-exchangeable groups, H^+^ and OH^−^ in the solution under acidic and basic medium. The possible reaction mechanism between the adsorbent and anionic IC molecules (^−^IC) are representing as follows:(CuAl-LDH/SWCNTs)^+^X^−^ + ^−^IC → (CuAl-LDH/SWCNTs)^+^·^−^IC + X^−^ (anion exchange)(CuAl-LDH/SWCNTs)^+^X^−^ + H^+^ → ^+^H·(CuAl-LDH/SWCNTs)^+^·X^−^ (under acidic conditions)^+^H(CuAl-LDH/SWCNTs)^+^X^−^ + 2IC^−^ → IC^−^·^+^H(CuAl-LDH/SWCNTs)^+^·^−^IC (anion exchange and electrostatic interaction in acidic conditions)where, X^−^ represents the anionic species in CuAl-LDH layers and ^−^IC is anionic IC molecules. Moreover, under basic conditions, OH^−^ are in excess in the solution which generates the negative charge on the surface of the adsorbent resulted in electrostatic repulsion forces develop between the anionic IC molecules and negatively charged CuAl-LDH/SWCNTs nanocomposite. The anion exchange also reduced due to the presence of excessive OH^−^ and there is a completion between OH^−^ ions and ^−^IC molecules for the same site. Thus, adsorption of the IC reduced in the basic pH conditions.^37^ Besides the electrostatic forces and anion exchange mechanism, some other forces such as van der Waals forces, π–π interaction, H-bonding, and physical adsorption are also responsible for the adsorption of the IC molecules onto the CuAl-LDH/SWCNTs nanocomposite as shown in Fig. 1. The SWCNTs sidewalls are hydrophobic in nature due to higher π election density of sp^2^ carbon atom which may interact with the IC molecules *via* hydrophobic interactions. Moreover, the π system of the SWCNTs in nanocomposite may interact with π bond (C=C) in the benzene ring of the IC molecules and establish the π–π interaction. The carboxylic and hydrophilic groups present on the CuAl-LDH/SWCNTs nanocomposite also form the H-bond with the nitrogen and oxygen-based functional groups presents in IC molecular structure.^1,7,11^ A schematic mechanism for the adsorption of IC molecules onto the CuAl-LDH/SWCNTs nanocomposite is shown in Fig. 1.

#### 3.2.2. Adsorption isotherms and thermodynamics

**Fig. 5:**
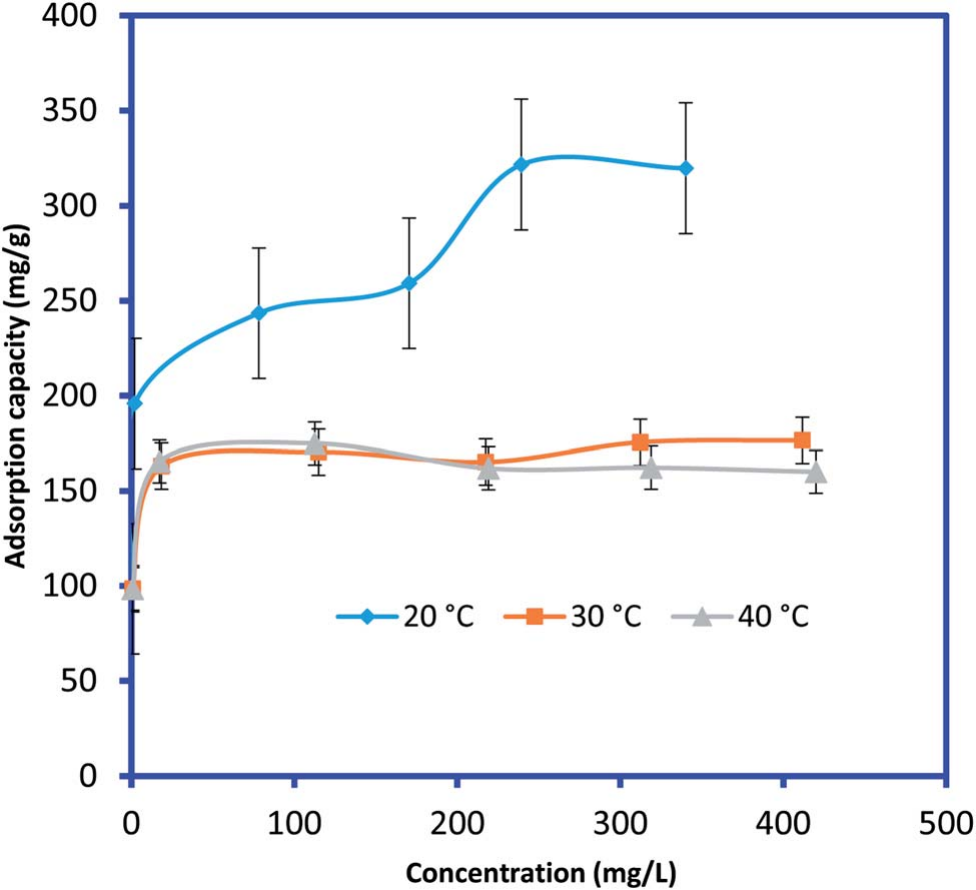
Effect of initial IC dye concentration and solution temperature on IC adsorption onto CuAl-LDH/SWCNTs nanocomposite (*V* 20 ml, time 6 h, *m* 0.01 g, pH 2).

**Fig. 6:**
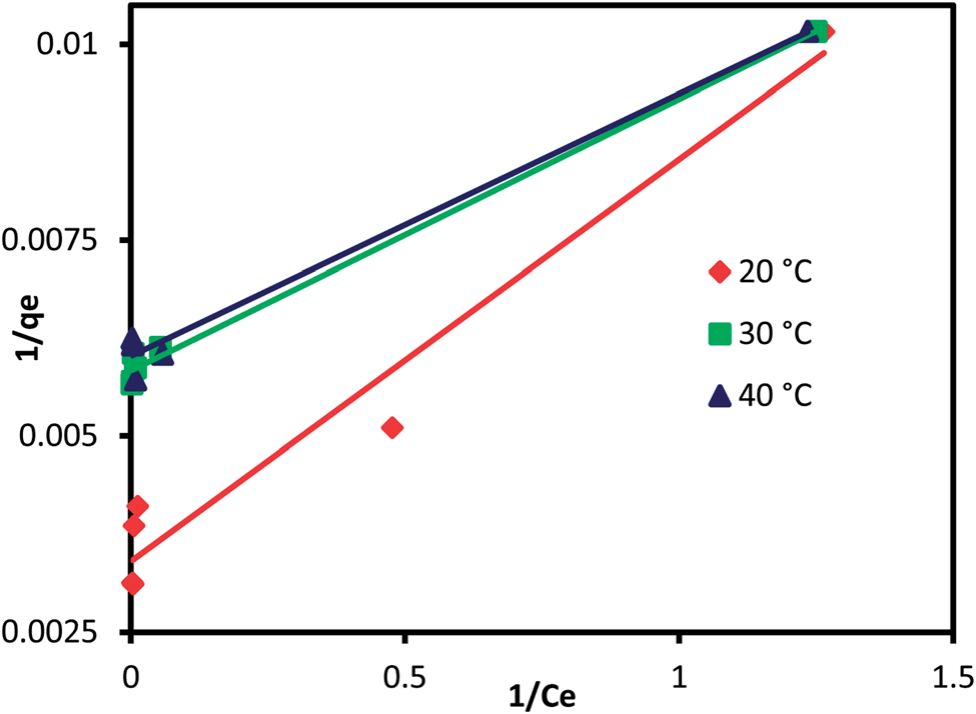
Langmuir isothermal plot for adsorption of IC onto CuAl-LDH/SWCNTs nanocomposite.

In order to determine the effect of IC concentration and temperature, the adsorption studies were performed at the concentration ranges from 50 to 500 mg L^−1^ and temperature at 20, 30, and 40 °C, respectively. The results are depicted in Fig. 5 and an increase in temperature has a negative impact on IC adsorption while the adsorption of the IC molecules rises with the increase in the initial IC concentration in the solution. The adsorption capacity of IC onto CuAl-LDH/SWCNTs nanocomposite decreased from 259.16 to 161.86 mg g^−1^ with a rise in solution temperature from 20 to 40 °C. The decrease in the uptake of IC may refer to the deformation of the adsorption sites on CuAl-LDH/SWCNTs nanocomposite and exothermic nature of adsorption process.^36^ Moreover, to find the effect of IC concentration and solution temperature on the interaction between the IC molecules and CuAl-LDH/SWCNTs nanocomposite, Langmuir^38^ and Freundlich^39^ isotherm models are applied to the adsorption data. The linear equation for the Langmuir and Freundlich isotherm models are as follows;2
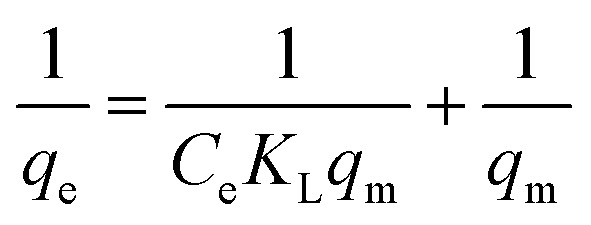
3
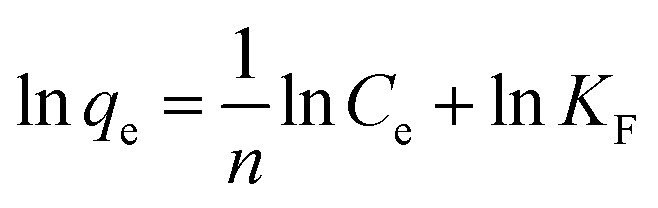
where *q*_m_ (mg g^−1^) is the CuAl-LDH/SWCNTs nanocomposite maximum monolayer adsorption capacity of IC molecules and *K*_L_ (L mg^−1^) is the free energy of adsorption. Both *q*_m_ (mg g^−1^) and *K*_L_ (L mg^−1^) are the Langmuir constant. *C*_e_ (mg L^−1^) is the IC equilibrium concentration. The Freundlich constant, *K*_F_ denote the adsorption capacity and *n* represents adsorption intensity. Fig. 6 and S2† shows the plots for Langmuir and Freundlich isotherms model applied for the IC removal by CuAl-LDH/SWCNTs nanocomposite, respectively. The parameters values calculated from their respective plots are tabulated in Table 1 and the obtained values for the parameters revealing that the adsorption of IC onto CuAl-LDH/SWCNTs nanocomposite followed the Langmuir isotherm model which suggesting the monolayer adsorption of IC molecules onto the CuAl-LDH/SWCNTs nanocomposite surface.^8^ The obtained *q*_m_ values at 20, 30 and 40 °C, are 294.117, 172.413 and 166.66 mg g^−1^, respectively, which are close to the experimental data. The adsorption capacity of the CuAl-LDH/SWCNTs nanocomposite for IC is higher than the previously reported adsorbents such as raw Brazil nut shells – 1.09 mg g^−1^,^40^ palm wood cellulose activated carbon – 1.85 mg g^−1^,^41^ bio-MnO_*x*_-C – 45.95 g/0.1 g,^42^ Fe/Mg-LDH – 55 mg g^−1^,^8^ chitin – 5.75 and chitosan – 71.82 mg g^−1^.^7^

**Table 1:** Isotherm parameters for the removal of IC onto CuAl-LDH/SWCNTs nanocomposite

Temp. °C	Langmuir isotherm model			Freundlich isotherm model		
	*q*_m_ (mg g^−1^)	*K*_L_ (L mg^−1^)	*R* ^2^	*K*_F_ (mg^1−1/*n*^ L^1/*n*^g^−1^)	*n*	*R* ^2^
20	294.117	0.666	0.9608	123.766	6.142	0.8475
30	172.413	1.657	0.9930	107.018	11.148	0.8500
40	166.66	1.818	0.9839	111.396	13.404	0.7000

The thermodynamic parameters values for the adsorption of IC onto CuAl-LDH/SWCNTs nanocomposite are calculated using the following equations.4
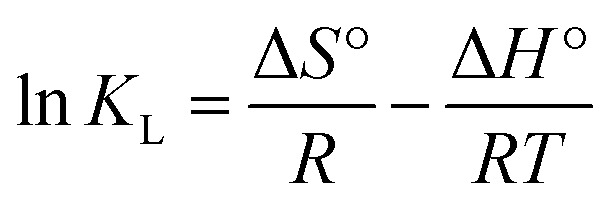

5 Δ*G*° = −*RT* ln *K*_L_

where, Δ*G*°, Δ*H*°, and Δ*S*° are the free energy change (kJ mol^−1^), enthalpy change (kJ mol^−1^), and entropy change (J mol^−1^ K^−1^), respectively. *K*_L_ is the Langmuir constant (L mol^−1^). A plot of Van't Hoff, ln *K*_L_*versus* 1/*T*, is shown in Fig. S3† and the obtained values for the thermodynamic parameters are tabulated in Table 2. As the negative Δ*G*°, IC adsorption onto CuAl-LDH/SWCNTs nanocomposite is spontaneous in nature.^42^ The value of Δ*H*° is positive, indicating the exothermic adsorption of IC and onto CuAl-LDH/SWCNTs nanocomposite. A positive value of Δ*S*° demonstrates an increasing randomness at the onto CuAl-LDH/SWCNTs nanocomposite IC solution interface during the adsorption process.^43,44^

**Table 2:** Thermodynamic parameters for IC adsorption onto CuAl-LDH/SWCNTs nanocomposite

Temperature (°C)	Δ*G*° kJ mol^−1^	Δ*H*° kJ mol^−1^	Δ*S*° J mol^−1^ K^−1^
20	−30.822		
30	−34.170	38.784	0.2383
40	−35.539		

#### 3.2.3. IC adsorption kinetics

**Fig. 7:**
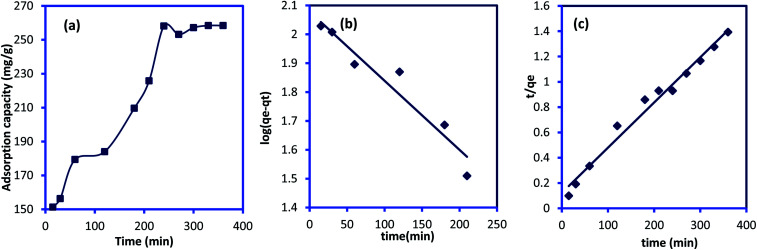
(a) Effect of contact time on IC dye adsorption onto CuAl-LDH/SWCNTs nanocomposite (*m* 0.01 g, *V* 20 ml, temp. 20 °C, conc. 300 mg L^−1^), and plot for (b) pseudo-first-order (c) pseudo-second order kinetic models.

The adsorption of IC molecules onto CuAl-LDH/SWCNTs nanocomposite at the varying contact time is shown in Fig. 7a. The uptake of IC gradually rises as the times increases and shows a fast adsorption at the initial 100 min. Thereafter, adsorption of IC onto CuAl-LDH/SWCNTs nanocomposite become slow because of complete anion exchange and saturation of all the active sites within 230 min. The initial fast removal of the IC was mainly due to the quick anion exchange and adsorption on the vacant site available onto the CuAl-LDH/SWCNTs nanocomposite. Thereafter, an adsorption–desorption equilibrium is established between the IC molecules in the solution and adsorbed on CuAl-LDH/SWCNTs nanocomposite surface.^36^ To get more information about the adsorption–desorption equilibrium and rate of IC adsorption, the pseudo-first-order,^45^ and pseudo-second-order^46^ models were applied to the obtained data. The linear equation for the pseudo-first order, pseudo-second order are:6
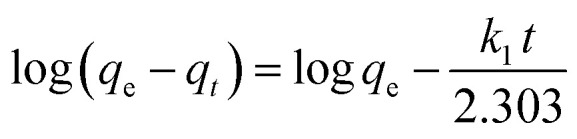
7
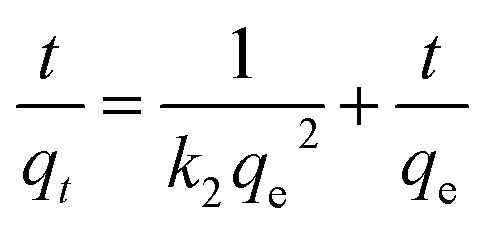
where *q*_*t*_ and *q*_e_ are the IC adsorption capacity (mg g^−1^) onto CuAl-LDH/SWCNTs nanocomposite at time *t* (min) and equilibrium, respectively. *k*_1_ (1/min) and *k*_2_ (g mg^−1^ min^−1^) represent the rate constant for pseudo-first-order and the pseudo-second-order. Fig. 7b and c show the respective plots for the applied kinetic models and the calculated values for each model are given in Table 3. The calculated values indicated that the adsorption of IC onto CuAl-LDH/SWCNTs nanocomposite followed the pseudo-second-order kinetic model because of higher *R*^2^ values (*R*^2^ = 0.98227) than *R*^2^ obtained from the pseudo-first order (*R*^2^ = 0.9314). Besides this, the calculated *q*_e_ for the pseudo-second-order model is much closer to the experimental *q*_e_ values which confirming the applicability of the pseudo-second kinetic model to the adsorption data.^44^ The diffusion mechanism of the IC molecules form the solution to the CuAl-LDH/SWCNTs nanocomposite has been investigated by applying the Weber and Morris intra-particle diffusion model to the kinetic data. The equation for Weber and Morris intra-particle diffusion model is:8
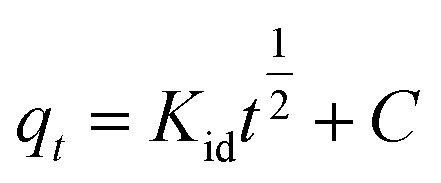
where *q*_*t*_ is the adsorption capacity at time *t*, and *K*_id_ represents the intra-particle diffusion rate constant (mg g^−1^ min^−1/2^). A plot for Weber and Morris intra-particle diffusion model is shown in Fig. S4.† The plot shows the multilinear porting for the adsorption of IC molecules onto CuAl-LDH/SWCNTs nanocomposite. The first portion indicates the fast adsorption of the IC through electrostatic forces which the second linear curve indicate the diffusion of IC molecules into the layered structure of CuAl-LDH/SWCNTs nanocomposite while the third portion showing the complete saturation of the adsorbent. This model suggested that the adsorption of the IC takes place on the surface as well as in the internal structure of the CuAl-LDH/SWCNTs nanocomposite.^47^

**Table 3:** Kinetic parameters for the IC adsorption onto CuAl-LDH/SWCNTs nanocomposite

*q* ^(exp)^_e_ (mg g^−1^)	Pseudo-first order model			Pseudo-second order model		
	*q* ^(cal)^_e_ (mg g^−1^)	*k*_1_ (min^−1^)	*R* ^2^	*q* ^(cal)^_e_ (mg g^−1^)	*k*_2_ (g mg^−1^ min^−1^)	*R* ^2^
258.14	119.784	5.527 × 10^−3^	0.9314	277.77	1.057 × 10^−3^	0.9822

## 4. Conclusions

A novel anion selective ion-exchanger CuAl-LDH/SWCNTs nanocomposite has been synthesized using the urea hydrolysis method. The results demonstrated the adsorption of anion IC molecules onto CuAl-LDH/SWCNTs nanocomposite is controlled by the solution pH, temperature, initial IC concentration in solution and reaction time. The highest uptake of the IC onto CuAl-LDH/SWCNTs nanocomposite was observed within 230 min at pH 2 and 20 °C. The adsorption of IC was exothermic in nature and monolayer adsorption capacity of CuAl-LDH/SWCNTs nanocomposite for IC reduced from 294.117 to 166.66 mg L^−1^ as the solution temperature rises from 20 to 40 °C. The mass transfer rate of IC from solution to CuAl-LDH/SWCNTs nanocomposite was controlled by the pseudo-second-order kinetic model. Hence, the intraparticle diffusion model confirm the deposition of IC at the out surface and inner layered structure of CuAl-LDH/SWCNTs nanocomposite. The comparative results revealed that CuAl-LDH/SWCNTs nanocomposite has a higher adsorption capacity for IC compared to the previously reported materials. Based on these results, we can conclude that CuAl-LDH/SWCNTs nanocomposite is an efficient adsorbent for the removal of IC from the wastewater.

## Conflicts of interest

There are no conflicts of interest to declare.

## Supplementary Material

RA-009-C8RA09562K-s001
