# Supplementary material for: Adsorption and anion exchange insight of indigo carmine onto CuAl-LDH/SWCNTs nanocomposite: kinetic, thermodynamic and isotherm analysis
Source: RSC Adv. 2019 Jan 2;9(1):560–8. doi: 10.1039/c8ra09562k (PMC9059263; doi:10.1039/c8ra09562k)
Supplement: RA-009-C8RA09562K-s001 [file RA-009-C8RA09562K-s001.pdf]

Electronic Supplementary Material (ESI) for RSC Advances.
This journal is © The Royal Society of Chemistry 2018

Adsorption and anion exchange insight of indigo carmine onto CuAl-LDH/SWCNTs nanocomposite: kinetic, thermodynamic and isotherm analysis

Noha Almoisheer,[a] F.A. Alseroury,[a,b] Rajeev Kumar,[c] M. Aslam,[d] M.A. Barakat[c,e*]

[a]Department of Physics, Faculty of Science, King Abdulaziz University, KSA

[b]Department of Physics, Faculty of Science, University of Jeddah, KSA

[c]Department of Environmental Sciences, Faculty of Meteorology, Environment and Arid Land Agriculture, King Abdulaziz University, Jeddah, 21589, KSA

[d] Center of Excellence in Environmental Studies, King Abdulaziz University, KSA

[e]Central Metallurgical R & D Institute, Helwan 11421, Cairo, Egypt

*Corresponding Author's Email: mabarakat@gmail.com

Phone: +96626400000, ex. 64821

Fax : +96626952364

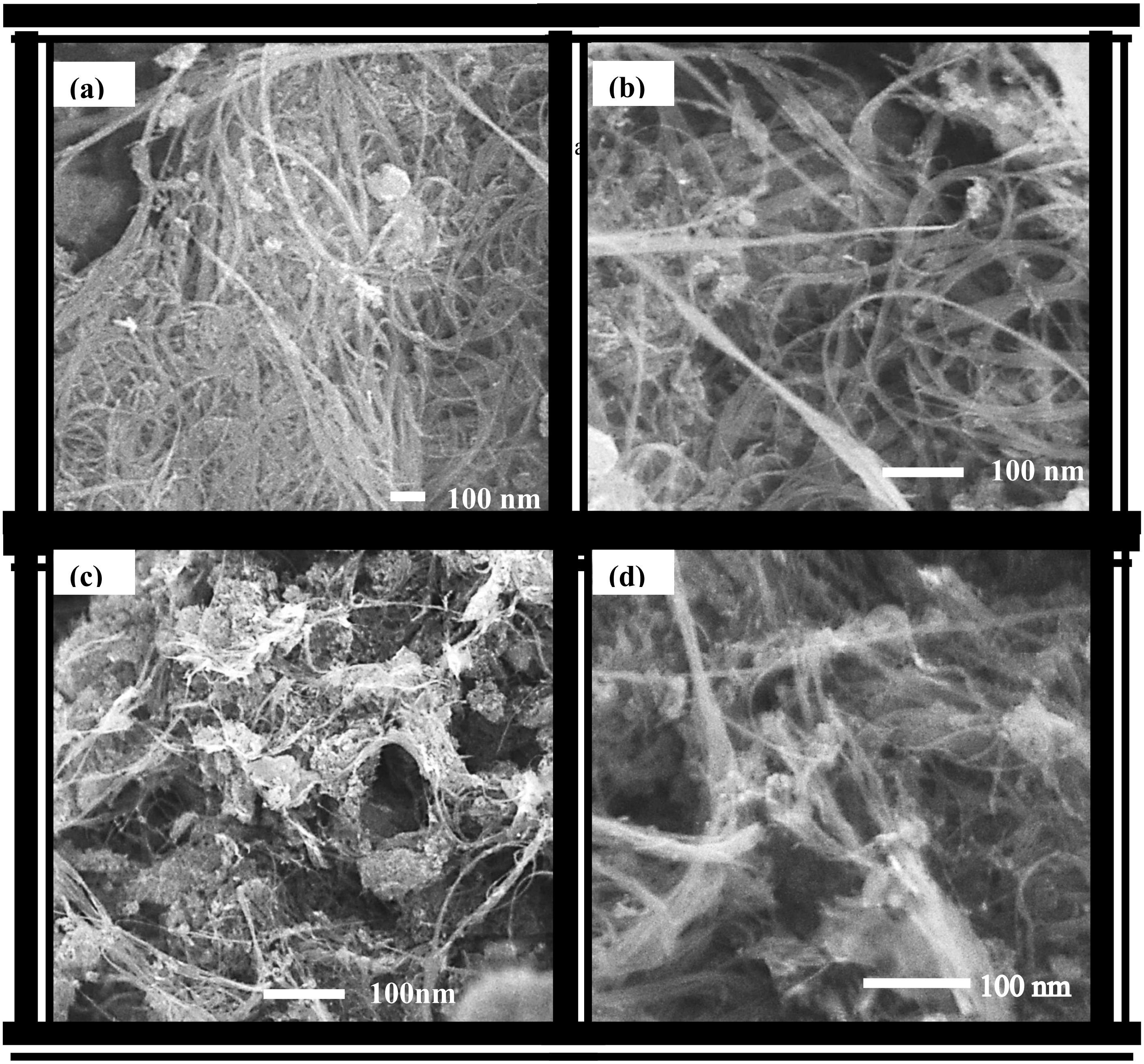

Fig. S1. SEM Images of (a,b SWCNTS) and (c, d) CuAl-LDH/SWCNTs nanocomposite

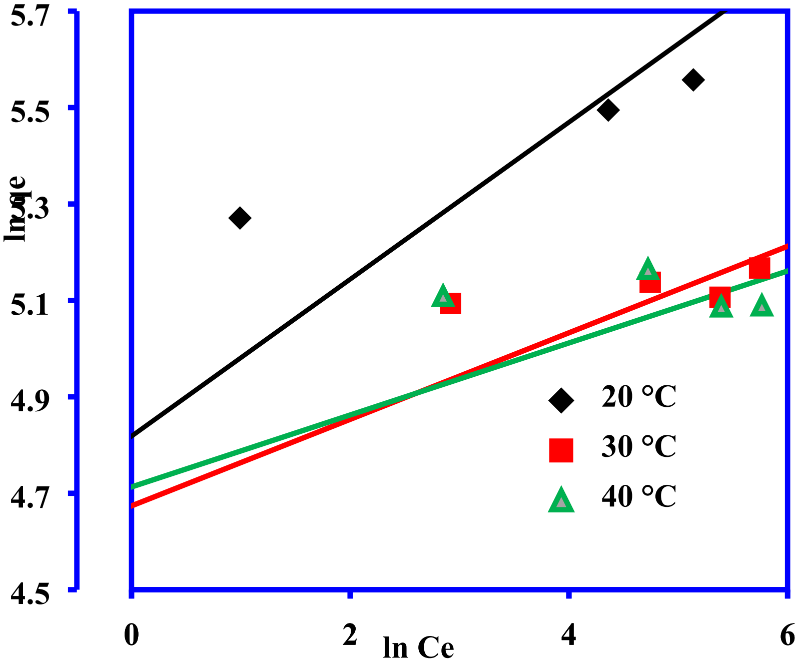

Fig. S2. Freundlich isotherm plot for adsorption of IC onto CuAl-DLH/SWCNTs nanocomposite

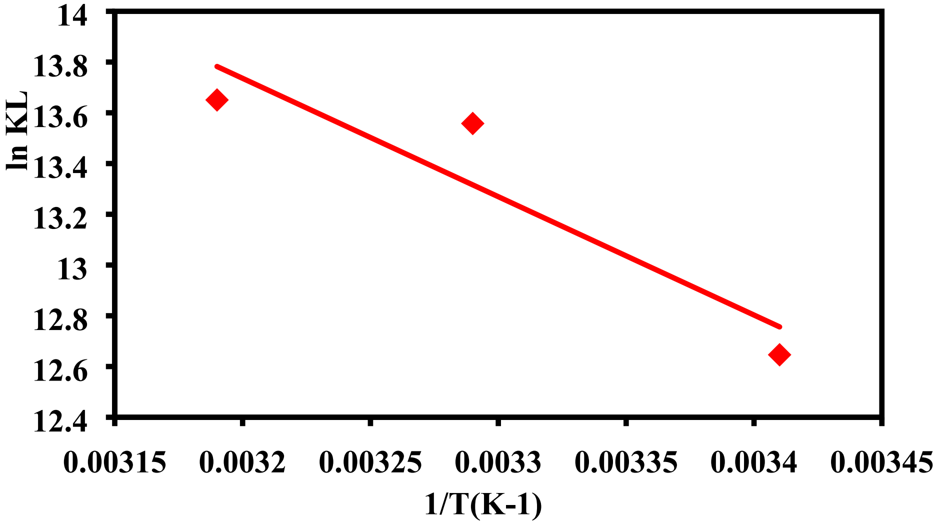

Fig. S3. Van't Hoff plot for the adsorption of IC onto CuAl-DLH/SWCNTs nanocomposite

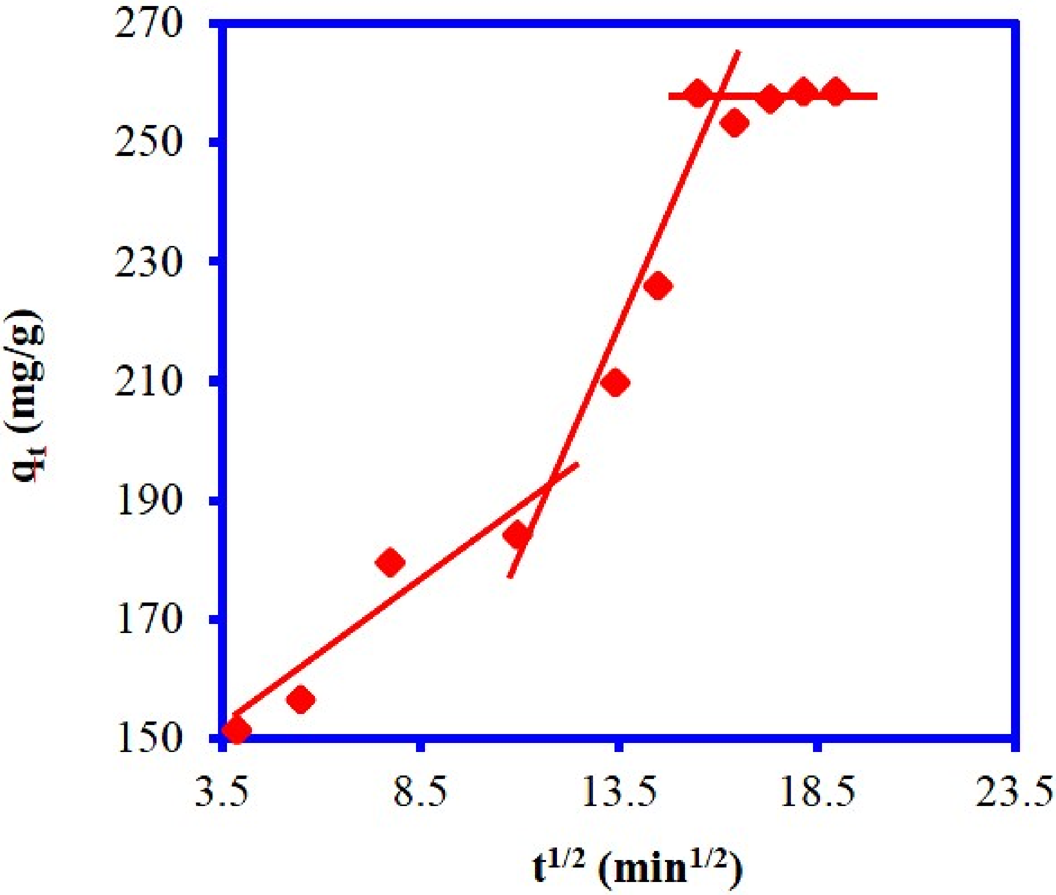

Fig. S4. Weber-Morris intraparticle diffusion for IC adsorption on to CuAl-DLH/SWCNTs nanocomposite
